# Taming age mortality in semi-captive Asian elephants

**DOI:** 10.1038/s41598-020-58590-7

**Published:** 2020-02-05

**Authors:** Jennie A. H. Crawley, Mirkka Lahdenperä, Zaw Min Oo, Win Htut, Hnin Nandar, Virpi Lummaa

**Affiliations:** 1 Department of Biology, University of Turku, Turku, 20014 Finland; 2 Myanma Timber Enterprise, Ministry of Natural Resources and Environmental Conservation, Yangon, Insein Township Myanmar

**Keywords:** Ecology, Physiology, Zoology, Ecology

## Abstract

Understanding factors preventing populations of endangered species from being self-sustaining is vital for successful conservation, but we often lack sufficient data to understand dynamics. The global Asian elephant population has halved since the 1950s, however >25% currently live in captivity and effective management is essential to maintain viable populations. Here, we study the largest semi-captive Asian elephant population, those of the Myanma timber industry (~20% global captive population), whose population growth is heavily limited by juvenile mortality. We assess factors associated with increased mortality of calves aged 4.0–5.5 years, the taming age in Myanmar, a process affecting ~15,000 captive elephants to varying degrees worldwide. Using longitudinal survival data of 1,947 taming-aged calves spanning 43 years, we showed that calf mortality risk increased by >50% at the taming age of four, a peak not seen in previous studies on wild African elephants. Calves tamed at younger ages experienced higher mortality risk, as did calves with less experienced mothers. Taming-age survival greatly improved after 2000, tripling since the 1970’s. Management should focus on reducing risks faced by vulnerable individuals such as young and first-born calves to further improve survival. Changes associated with reduced mortality here are important targets for improving the sustainability of captive populations.

## Introduction

We are currently facing widespread population declines around the world across taxa, with more and more species threatened by human activity. It is vital to monitor populations in order to limit and mitigate these anthropogenic effects^1^. The global Asian elephant (*Elephas maximus)* population has halved since the 1950s, and its range has shrunk to 5% of its original size, leaving it endangered^2^. Uniquely however, ~15,000 Asian elephants live among humans in captive and semi-captive conditions, which constitute approximately 24–29% of the remaining global population^2,3^. Whilst the conservation priority is to protect the continuously shrinking wild population, optimal management of these semi-captive populations has also become an increasingly important tool for maintaining a viable global population^4,5^.

The largest of these semi-captive populations is the timber elephant population of Myanmar, which consists of over 5,000 individuals, and is in fact larger than the wild population^6^. Over half of this population is owned and managed by the state-run Myanma Timber Enterprise (MTE), which allows consistent management practices to be applied across the country^7^. Historically, the Myanma population has relied on capture from the wild, but routine capture has been banned for over two decades^8^, and the semi-captive population may not be self-sustaining in its current state^4^. Recent individual-based projection models of longitudinal birth and death records in this population suggested that population growth is limited most by low birth rates and high juvenile mortality^4^ with over 25% of calves reported to die before the age of five^9^. Minimizing juvenile mortality was suggested as the most obtainable and promising management target for this population^4^. High mortality during the first two years of life follows typical biological patterns seen across many species, including African elephants^10,11^. However, a second peak in mortality occurs around age four in the MTE elephants. This mortality peak could be linked to the taming procedure of these working animals that occurs at this age^9^, in which case this period could be an important target for management. Higher mortality at this age could be caused by the taming itself or changes that accompany this process such as separation from the mother, weaning and translocation.

Taming is currently a necessary process for the Myanmar population of timber elephants, to ensure a safe and effective working environment. These large animals can pose great risks to their handlers, or mahouts^12^, and have been reported in the past to cause 10–20 mahout fatalities per year within the MTE^7^. During taming, an elephant is introduced to human contact, their behaviour is controlled and they are taught to respond to various commands. Taming is a process that occurs to varying degrees for the ~15,000 semi-captive Asian elephants living among humans worldwide, in logging, transport, tourism or other industries. Although there has been a recent trend towards protected contact handling (where a barrier is maintained between animal and handler) for captive elephants, especially in western zoos that hold a further ~1,000 elephants, this concerns one tenth of all captive elephants at most^13^, and much debate and taboo surrounds taming methods even within European zoos^14^. Therefore, as a potential source of mortality within our control, it is vital to acknowledge and understand the dynamics underlying mortality during taming ages in order to minimize mortality risks.

Several key factors could influence an individual’s probability of survival during taming. Mortality may either be directly caused by taming, for instance due to injury or accident, or indirectly, due to infectious diseases, parasites, weakness from training or a combination of many factors. Deaths caused directly by taming are more likely symptoms of the taming process itself, and to suggest modifications or changes to this traditional process is a complicated cultural issue outside the scope of this paper. Indirect deaths, on the other hand, are more linked to individual elephant traits, which we primarily focus on here. For example, indirect mortality may depend on body condition, as individuals with low body condition can be immunosuppressed, and therefore more susceptible to e.g. parasites or disease^15,16^. For example, the effect of stress on factors affecting a calf’s susceptibility to diseases such as the Elephant Endotheliotropic herpesvirus (EEHV), which currently threatens both African and Asian elephant calves globally^17^, is poorly understood and taming stress could exacerbate disease susceptibility. Calf traits such as body size and condition in turn vary with many factors such as a calf’s environmental and early life conditions, their sex, age and the maternal care they receive. First, males experience higher juvenile mortality than females in many species^18,19^, which could be due to higher energetic demand in species where males are larger, such as in Asian elephants^20^. This applies when mortality pressures are size discriminate due to, for example, limited resources rather than due to disease or predation^19,21^. Males are known in our study population to have higher mortality in early life and adulthood^9,22^, and also more parasite related deaths^23^. There are also sex differences in taming approaches because tusked males are seen to pose more risks than females that lack tusks, even at this age when tusk size is limited. It is however currently unknown whether stress results in sex differences in mortality during taming, a time of conflict during which the larger size of males could instead be an advantage. Second, maternal factors are important to calf survival during high-stress periods, especially in species with slow life histories such as the Asian elephant, where gestation lasts nearly two years and calves are completely dependent on their mothers for the first two years and suckle for several more^3^. Consequently, first born calves with inexperienced mothers, and those born to ageing mothers with decreased body condition, have generally been found to experience higher juvenile mortality^9^. Another factor with the potential to influence calf survival is their mother’s birth origin, as females caught from the wild and not born in captivity have previously been found to show compromised reproduction, survival themselves and calf survival compared to mothers born in captivity^4,24,25^. However, whether similar impacts extend to their calves during the critical period of taming is unknown, as are the effects of maternal investment. Third, taming age varies across the countries where captive elephant populations are common, but how calves of different ages cope with taming and consequently how this affects their mortality risk is unknown. Finally, it also remains unclear whether taming-age mortality of calves has changed over time as veterinary care, taming practices and general management have shifted in the modern day^26^.

Here, we investigate calf characteristics related to survival during the ages of taming in the Asian elephant. We investigate the importance both of calf traits, such as when they were born, their sex and age at taming, as well as mother traits such as their mother’s age, origin and past maternal experience, to pinpoint factors which could indicate calf susceptibility to mortality risks during this period, and to document how taming-age mortality has varied over time. To this end, we use unique longitudinal records of births and deaths maintained by veterinarians overseeing taming events over more than four decades. We first quantify the survival of 2,962 semi-captive working elephants over their first ten years, and compare mortality rates of these semi-captive elephants to previously published mortality rates of wild African elephants^11^ not subject to taming in order to assess whether deaths in this semi-captive population are likely linked to their management and therefore may be preventable (similar data on wild Asian elephants is not to our knowledge available). We next determine the mortality of 1,947 elephants during taming ages (4.0–5.5 years) according to key demographic traits, in calves born across Myanmar between 1970–2013.

## Methods

### Study system

This study focuses on elephants of the Myanma Timber Enterprise (MTE), who own and employ over half of the semi-captive elephants in Myanmar (~3,000 individuals), as draught animals to transport felled logs through the forest. The MTE keeps detailed records of all of their elephants, enabling us to monitor the life events (e.g. birth and death) of individual animals^4,24,27^. Each elephant has a unique ID number permanently marked on their rump, and veterinarians and other authorised personnel maintain a logbook throughout their life recording important demographic and health events. These include exact birth and death dates^24^, cause of death determined by post-mortum^23^, location, and maternal information^28^. Here, we use this information to investigate factors associated with mortality risk during taming age for captive-born calves. The MTE limits work for each elephant in terms of hours and tonnage according to their age, condition, size/experience and the season of the year, and all are retired by the age of 55. The MTE elephants live in semi-captive conditions; they roam in the forest at night to forage and socialize with conspecifics including wild elephants, where they mate unsupervised. Current management rests females from mid-late pregnancy until their calf reaches 1.5 years, when they return to light work with their calf at heel. Normally, calves have limited contact with humans (although it has been recommended in recent years for mahouts to start to handle calves from a young age) and they suckle at will from their mother until they reach around four years. At this point, they are weaned, separated from their mother and start the taming procedure.

#### The taming procedure

The traditional taming procedure takes place for approximately one month at a designated taming camp, with usually no more than four calves tamed in the same camp. To minimize heat related stress, taming always takes place in the cold season (starting late November/early December) for captive-born calves. Calves are usually tamed in the cold season of their fifth year, or when they reach approximately five feet two inches (157.5 cm), and special permission must be granted for early taming (e.g. for some calves with younger siblings competing for milk), or later taming (e.g. for orphaned calves). The taming procedure is conducted and supervised by a group of around eight mahouts, two or more experienced head mahouts per elephant, known as *sin-gaungs*, and one regional head mahout per camp, known as a *sin-oke*. The taming site is chosen to be shaded, level, and have access to plentiful fodder and water (for more information see cradle method in^29^). Staple fodder (e.g. bamboo leaves, paddy and broom grass), gathered from the forest by the mahouts is in constant supply, and supplements (e.g. rice, banana stem, tamarind with salt) are provided daily during taming. An MTE veterinarian stays in the camp and attends to the calves for the duration of the taming period, and each calf is taken for a walk every morning to bathe and drink from a natural water source (~30 minutes- three hours). Each calf experiences multiple bouts of ~45 minute training periods each day, usually in the morning and evening. During these training periods, the team of mahouts surround and rub the elephant, whilst singing a traditional *shaw-pike* song (derived from an ancient hindi song) to the calf to calm and familiarize them with their voices and contact. For around the first week of the taming process, the calf’s movement is restricted by a breast band and ropes, to reduce the risk of accident for the calf and mahouts. Depending on the mahouts involved and the calf’s temperament, behaviour is controlled through both positive and negative reinforcement, rewarding good behaviour and punishing aggression. The restricted movement and punishment are considered stressful for the elephant, and welfare advocates therefore generally express concern over both the psychological and physical effects of the process^14^. Gradually, as the calves come to accept mahout contact, they are allowed to move around more freely, and eventually are released to forage in the forest during the day after around three weeks, and both day and night after around four weeks. The intense taming period is usually complete in four to six weeks, though training continues in the following months and years until each elephant is enrolled as a mature working animal at age 17^30^.

### Data selection

We first performed a survival analysis for 2,962 captive-born elephants born between 1970 and 2009, from which we plotted a survival curve displaying trends in mortality over the first ten years of life for all individuals with known sex, birth and death/censorship date. We then investigated the traits underlying taming-age mortality (4.0–5.5 years, see below) using records of 1,947 elephants born in captivity between 1970–2013 who survived to age four, with known birth date, origin, sex, location, mother’s origin, mother’s age at calf birth, past calving information and calf date of death or censoring. All statistical analyses were conducted in R version 3.5.3^31^. We assessed the significance of a term using likelihood ratio tests with the Chi-squared distribution, the significance of levels within a term using Tukey’s post-hoc test, and we retained terms considered to be biologically important in the model. All data and relevant R code can be found in the Supplementary Information.

### Statistical analyses

#### Juvenile survival probability

We investigated juvenile survival through a survival analysis using the *survfit* function of the *survival* package^32^, with an explanatory variable of sex (binomial: n = 1,449 female, n = 1,513 male). From this, we plotted a survival curve, including individuals from their birth until their point of departure (either due to death: n = 1,030, or censorship/surviving until the end of the study: n = 1,932), from which we display the first ten years of life. We also used this survival model with an integer age term to estimate age specific mortality rates, calculating survival at year t as cumulative survival at t +1/cumulative survival at t and subtracting from 1 to retain mortality rather than survival rates. We then compare these age specific mortality rates over the first ten years in calves from the MTE population to those of wild African elephants in the Amboseli population published by Moss *et al*.^11^. Such data on wild Asian elephants were unavailable, but we deem the life history of the two species similar enough for suitable comparisons with similar: lifespan (max. 70–75 vs 76 years), inter-birth interval (average 4.5 vs 4.2 years), primiparity (average 14.1 vs 13.4 years) and weaning age (three-five years vs ~four years) in African and Asian elephants respectively^33–36^.

#### Influence of calf and maternal traits on taming age mortality

Next, we assessed how different traits affected a calf’s probability of survival during the ages of taming. We fitted a *binomial glmer* model with a logit link function from the *lme4* package^37^ with the response variable of whether or not a calf had died during taming ages (binomial:0/1). We defined taming age as between four and 5.5 years to include deaths both at the usual taming age (four years), and those in the months following. We included only individuals who had survived until the age of four and excluded those who had last been seen and censored under 5.5 (<2% of sample).

Fixed effects included calf sex (binomial: n = 963 male; n = 984 female), birth month (integer: 1–12 corresponding to January-December), birth decade, with calves born between 2010–2013 grouped in the 2000 decade (four level factor: n = 404 1970–79; n = 680 1980–89; n = 452 1990–99; n = 411 2000–13). We also included maternal age as a continuous variable with extreme values restricted to the 98^th^ percentiles (range 14–54 years), scaled and centred around the mean (mean = 31 years), and mother’s origin (binomial: n = 952 captive; n = 995 wild). Calves with captive born mothers were assigned a maternal origin of 0 and those with wild caught mothers 1. We included an interaction between this term and a log transformed continuous variable of time since mother’s capture at their calf’s birth (ranging from 0–48 years for wild caught and set at 0 for all captive born mothers), to account for the negative impacts of capture on calf survival lessening over time (see^24,25^). We also included a fixed effect of calf birth order with calves without siblings given a parity of 1. We split this variable into three categories (n = 630 1 = 1^st^ born; n = 819 2 = 2^nd^–3^rd^ born; n = 498 3 = 4^th^–11^th^ born) to counter the correlation with mother’s age (0.60 [0.57, 0.62]), and the resulting variance inflation factors of these terms were <2 and therefore deemed appropriate to include in the same model (according to^38^). We included a random effect of location (eight level factor) to adjust for any spatial variation in environment and survival. We combined divisions with few observations based on proximity and geographical features, with Chin (n = 2), Rakhine (n = 24), Tanintharyi (n = 4) and Shan (n = 115) states grouped based on high elevation, and Ayeyarwaddy (n = 61) and Yangon (n = 3) based on proximity. The rest of the divisions included were Bago (n = 284), Kachin (n = 65), Magway (n = 316), Mandalay (n = 116), Nay Pyi Taw (n = 83) and Sagaing (n = 874).

The use of birth month in its integer form is unusual, but we deemed it to be appropriate in this context as a proxy of calf age relative to the static taming period at the end of the year (November/December). For example, a calf born early in the year is always older during taming than those from the same cohort born towards the end of the year. We did not include a random effect of mother ID as around half the mothers were not repeated, i.e. only had one offspring in the dataset. We tested a quadratic term of mother’s age in case impacts were seen at the extremes, but this did not improve model fit and was not included in the final model (see Supplementary Table S1 for these effects).

## Results

### Juvenile survival probability

**Figure 1:**
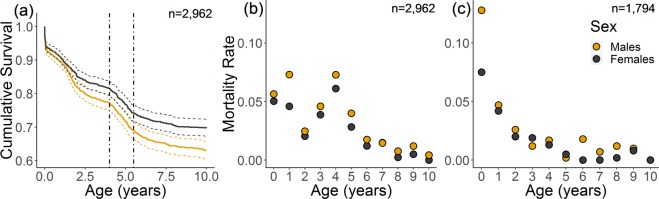
Survival of semi-captive working (**a**,**b**) and wild African (**c**) elephants in the first ten years of life as (**a**) cumulative survival and (**b**,**c**) age specific probability of death. Coloured dotted lines in (**a**) show confidence intervals and vertical black lines indicate taming ages (4.0–5.5 years). Mortality rates in (**b**) were deduced from a survival analysis with age as an integer, and in (**c**) were taken from Moss *et al*.^12^.

We first investigated calf survival for the two sexes over the first ten years of life. Males had generally lower survival at all ages, but both sexes showed a notable drop in survival around the taming age of four years **(**Fig. 1a**)**. Age-specific mortality increased by more than a half from the age of three until the age of four in the MTE elephants, rising by 55% and 66% for males and females respectively, matching and in females even exceeding, the high infant mortality rates seen in the first two years (Fig. 1b**)**. We then compared this acceleration in mortality at age four to rates published by Moss *et al*.^11^ from the Amboseli population of wild African elephants. These wild African elephants showed notably similar mortality patterns to the working elephants in our study population, with high infant mortality in the first two years of life and relatively stable lower mortality after the first two years up to age ten. However, in these wild elephants that are not subjected to taming, there was no evidence of the peak in mortality at age four seen in the semi-captive working elephants (Fig. 1c).

### Calf mortality according to calf and maternal traits

**Figure 2:**
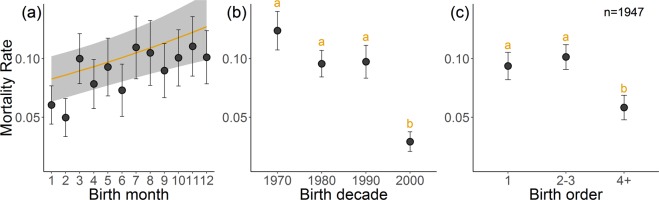
Calf mortality at taming ages depending on their (**a**) birth month, (**b**) birth decade and (**c**) birth order. Points show raw data, yellow line shows predicted values and letters indicate statistical separation of levels according to Tukey’s *post-hoc* tests. Predicted line is taken from a simplified model excluding random effects, and predictions are based on female elephants of birth order 1, born to a 30 year old mother in the 1990’s. Error bars and shaded areas show two standard errors.

Of the 1,947 calves included in our analysis, 171 had died during the ages of 4.0–5.5. These deaths could be interpreted as either directly linked to taming, or indirectly, such as those due to taming stress, infectious disease, parasites, or exhaustion (see Supplementary Information for causes of death and Supplementary Fig. S1 for distribution of deaths throughout the year). We assessed whether a calf’s chance of mortality during these ages depended on traits such as their sex, birth month or birth decade, the results of which are shown in Table 1. Although males displayed generally higher mortality (Fig. 1a), sex did not significantly affect a calf’s chance of mortality specifically during taming ages (χ^2^_1_ = 1.23, p = 0.27). A calf’s chance of mortality was positively related to their month of birth (χ^2^_1_ = 4.19, p < 0.05), with calves born in December, who were relatively younger at the onset of taming, having 1.7 times higher mortality than those born in January who were relatively older (Fig. 2a). Calf mortality also depended on their birth decade (χ^2^_3_ = 20.5, p < 0.001), with those born after 2000 having significantly lower mortality than those born in the three decades before (Fig. 2b shows statistical differences between groups). Calves born in the seventies had over three times higher mortality than those born after 2000 showing taming-age mortality has markedly reduced recently. We also investigated how maternal traits affected a calf’s likelihood to die during taming years. Calves of wild caught mothers who had been caught recently were slightly more likely to die during the taming age, as were calves of older mothers but these effects were not significant in this sample (Χ^2^_1_ = 1.86, p = 0.17; Χ^2^ = 3.68_1_, p = 0.06 respectively). However, calves who had lower parity and therefore less experienced mothers, were significantly more likely to die during the taming age (Χ^2^_2_ = 10.86, p < 0.01), with first born calves having 1.6 times higher chance of mortality than a fourth or later born calf (Fig. 2c shows statistical differences between groups).

**Table 1:** Glmer output of the effect of calf and maternal traits on survival at taming age (4.0–5.5 years).

Fixed effects	Estimate ± SE	z-value
(Intercept)	−2.11 ± 0.49	−4.36*
Sex (Male)	0.19 ± 0.17	1.12
Month Birth	0.05 ± 0.02	2.06*
Birth Order (2^rd^−3^rd^ born)	−0.08 ± 0.20	−0.39
Birth Order (4^th^ + born)	−0.84 ± 0.29	−2.89*
Mother’s Age	0.23 ± 0.12	1.94
Mother’s origin: Time since capture	−0.09 ± 0.07	−1.37
Birth Cohort (1980–89)	−0.26 ± 0.21	−1.25
Birth Cohort (1990–99)	−0.21 ± 0.23	−0.91
Birth Cohort (2000–13)	−1.37 ± 0.34	−4.02*
Random effects	**Variance **±** SD**	
Location	1.09 ± 1.05	

Estimates are expressed on the logit scale. The * symbol indicates statistical significance (p < 0.05) and : represents an interaction. Reference sex is female, birth order is first-born, and birth cohort 1970–79, n = 1,947.

## Discussion

More than a quarter of Asian elephants in the world today are semi-captive, living in human care, and yet even the largest of these populations, the ~5,000 timber elephants of Myanmar, is not currently self-sustaining^39^. One of the most important factors limiting this population is high juvenile mortality^4^, a substantial proportion of which is likely linked to the taming procedure. For now, the taming process is necessary in this population for the safety of those working so closely with these dangerous animals^12^, but it is a source of human-elephant conflict which can be managed. Here, we show that mortality peaks around the taming age of four, rising by over 50% compared to age three, and it appears this is a symptom of their management, likely the taming procedure, as a similar peak is not observed in wild African elephants^11^. We pinpoint traits associated with increased mortality at these ages, which can be used to adapt management to protect vulnerable individuals.

Males generally experience higher juvenile mortality in this population^9^ and past studies of Asian elephants found males to be more at risk during human-elephant conflicts such as poaching and crop protection, but it is unknown whether differences exist during taming ages^3,35^. Males are assumed to pose more risk during the taming process (due to their larger size^40^ and tusks), and therefore one could expect harsher treatment^41^. However, our study showed that males were no more likely to die than females during the taming ages of 4.0–5.5. It is possible that sex becomes less important at times of high mortality. For example, it has been shown in the past that sex differences are also negligible during the year following capture in this population during which mortality rates are very high^24^, and primiparous mothers have been shown to suffer high losses of both sons and daughters in African elephants^11^. It is also likely that taming is a time when a larger body size is actually beneficial for survival, so this may explain why the sex difference is lessened at these ages.

Mortality risk often depends on an individual’s body size or condition, such as deaths due to infectious diseases, exhaustion, parasites or gastro-intestinal issues, which together made up over a half of taming age deaths (see Supplementary Information). There are many factors that could be interpreted, with caution, as proxies for an individual’s body size or condition. Calves born earlier in the year, who were therefore older during the taming period at the end of the year, and therefore generally larger^40^, were significantly less likely to die. Furthermore, offspring body size has been shown to correlate with birth order in other species^42–44^ including African elephants^45^, with first born individuals often being smaller. Calves in our study born to less experienced mothers were significantly more likely to die during taming ages, likely reflecting calves in worse condition. This is consistent with past findings in this population that first time mothers also had more still births and lower overall calf survival up to age five^9^, and also findings from wild African elephants that calves with low parity were more at risk^11^. Calves of older mothers could also be expected to show higher mortality for this reason, due to the senescence of older females^46^, however we found that calves born to older mothers experienced slightly higher mortality during specific taming ages, but this effect was not significant. This is consistent with recent findings in this population that maternal age does not necessarily dictate survival in the first five years, but rather their overall lifespan^47^.

There are many other factors that could be influencing mortality around the time of the taming process, other than taming itself. It is possible that social influences could be driving differences between individuals, with some coping better with being separated from their mother than others. For example, calves naturally separate themselves from their mothers over time and males stray more than females^36^, so the reduced mortality difference between males and females at taming age compared to other ages and the higher survival in relatively older calves could be linked to a smoother and more natural separation from their mother. Furthermore, mortality around the taming age of four may be linked to weaning as calves are separated from their mother at this time, and mortality in some species can increase following weaning due to dietary adjustments^48^. However, elephant calves begin to eat solid food at three to six months^3,49^, and by four years are reasonably practiced at foraging with both Asian and African elephant calves seen to wean around this age in the wild^34,35^. To reduce the possibility that the increase in mortality observed at four in the working elephants is a general weaning-age pattern, we compared our findings to data available on wild African elephants not subject to taming (similar data on wild Asian elephants is to our knowledge not available). If the increase in mortality was due to weaning, we would expect to see a similar mortality peak in the wild population of Amboseli elephants^11^, but this is not the case despite notably similar mortality patterns in the two populations at other ages.

It is also possible that certain diseases prevalent in juvenile elephants, such as EEHV, could be contributing to age-specific mortality around 4.0–5.5 years, either independently of, or exacerbated by, taming. Although only two of the 171 calf mortalities studied here were officially attributed to EEHV, diagnosis has likely been under-reported especially in the past^17,50^. EEHV deaths have been reported to peak between 0–4 years of age generally^51^ and in Thai semi-captive elephants, the median age of infection has been reported lower at <2.5 years^17^, although this could be due to transport and weaning at young ages in the Thai tourism industry. However, we do not expect this disease to be the driving force behind the dramatic increase in mortality at the age of four seen in Fig. 1b independently of taming, although the risk of infection could be exacerbated by taming. Most deaths in calves aged 4.0–5.5 years occurred in the months immediately following taming (January onwards), in contrast to deaths clustering towards the end of the hot season (April-May) at other ages (see Supplementary Fig. S1). This makes it likely that the mortality peak is linked to the taming period, whether directly or indirectly. It is important to reiterate that we do not discriminate between direct and indirect impacts of taming in this study, so any disease or environmental effect likely to disproportionately impact stressed animals may contribute to the increase in mortality risk during taming. Furthermore, although both logging schedules and variation in climate likely impact general mortality in this population, they are unlikely to specifically affect taming-age elephants as calves are not involved in logging operations until >17 years, nor would they explain the change in monthly distributions of deaths seen in Supplementary Fig. S1^52^.

In terms of elephant management, Myanmar holds promise for future conservation, with the largest area of remaining natural habitat and ~5,000 semi-captive elephants of whom over half are centrally managed by the government who encourage breeding, factors often lacking in other countries^6,7,53^. The MTE have measures in place to minimize harm during taming, such as having a vet present for the whole period, daily walks, and more recently the gradual introduction of calves to human contact from the age of three months^29^. Promisingly, taming age mortality substantially decreased for calves born after 2000, consistent with reports of improved elephant treatment in the last two decades in this population^26^, and with more attention to individuals highlighted here as vulnerable, further improvements may be possible. It has been previously documented that calves of wild-caught mothers generally show increased infant mortality compared to calves of captive-born mothers in this population, especially in the year following their mother’s capture^25^. Although the chance of mortality at taming ages again reduced with increased time since mother’s capture, this trend and the effect of mother’s origin were not significant, which is encouraging considering over 40% of individuals in this population, across the study period, originate from the wild^24^.

Our interpretation that mortality differences according to birth order and calf age are linked to body size or condition has complex implications, as it concerns not only animal welfare, but also human safety. There is reluctance among elephant handlers to tame larger calves, as they pose more of a physical threat during times of close contact like taming, and the procedure can take longer, requiring more resources and money, and perhaps harsher measures^41^. Furthermore, the longer a calf stays with its mother, the longer she is kept from certain work tasks. Delaying taming would therefore require financial subsidies, which could be a major barrier in this population primarily driven by economic restraints rather than conservation, although there has been much focus recently on the latter^6^. The taming age of four in MTE elephants is already later than in other semi-captive populations, such as in Nepal, India and Thailand where taming generally occurs at age three^54–56^. Unfortunately, no comparable data exists from these populations on mortality risks during the taming process, with our study, to our knowledge, providing the first published estimates. We invite more studies into the effects of taming on Asian elephants across their range, as there are substantial differences between countries and even within countries that lack central management, and information from different perspectives could help us to further understand, and reduce, drivers of mortality. We also highlight the need for more study into population dynamics of wild Asian elephants to better inform comparisons to natural demographic parameters, which are for the moment largely lacking.

Now is an important time to address issues regarding taming, with largescale shifts within elephant management worldwide making change possible, to boost the survival of calves that are particularly vulnerable to taming risks^26,57^. The steep declines in mortality during taming in the MTE elephants over the last two decades are promising for the future of human-managed populations that are, for the moment, reliant on a degree of taming. In Myanmar, practices are changing, and managers sometimes recommend delayed or softer taming for certain female or orphaned calves. We recommend managers and those involved in taming worldwide consider adopting similar practises to these which have been coupled with reduced mortality, as well as considering the greater vulnerability of calves born to first-time mothers and those younger at the onset of taming. We urge for more studies across other populations and species to identify traits underlying mortality differences during periods of stress, be it due to anthropogenic or natural stressors, to inform management priorities.

## Supplementary information

Supplementary Information.

Supplementary Information2.

Supplementary Information3.

Supplementary Information4.

Supplementary Information5.

Supplementary Information6.

Supplementary Information7.

## Data Availability

All data and corresponding R code relevant to the analysis is included in the Supplementary Information.
