# Supplementary material for: Taming age mortality in semi-captive Asian elephants
Source: Sci Rep. 2020 Feb 5;10:1889. doi: 10.1038/s41598-020-58590-7 (PMC7002507; doi:10.1038/s41598-020-58590-7)
Supplement: Supplementary file 1 — Supplementary Information. [file 41598_2020_58590_MOESM1_ESM.pdf]

Taming age mortality in semi-captive Asian elephants

Jennie A. H. Crawley, Mirkka Lahdenperä, Zaw Min Oo, Win Htut, Hnin Nan Dar, Virpi Lummaa

Supplementary Information

Causes of Death

We loosely group causes of death similarly to Lynsdale et al. (2017), into *parasites* (total n=28) : parasites (1), worms (8) thut (3), bots (8), liver fluke (2), filariasis (6), *infectious diseases* (total n=30): anthrax (7), septicemia (7), herpes virus (2), tetanus (3), lung infection (2), pneumonia (9), *gastro-intestinal* (total n=14): enteritis (2), constipation (5), diarrhea (5), food poisoning (2), *exhaustion* (total n=22): general weakness (19), heat stroke (3), *injury* (total n=16): fall (12), suffocation (1), strangled (3), or *other* (total n=61):, heart attack (2), joint illness (1), liver abscess (1), sudden death (1), taming stress (33), unknown (15), anaemia (3), unknown disease (3), fat embolism (2).

Table S1. Glmer output of the effect of calf and maternal traits on survival at taming age (4.0-5.5 years) including a quadratic mother's age term. Estimates are expressed on the logit scale. The * symbol indicates statistical significance and : represents an interaction. Reference sex is female, birth order is first-born, and birth cohort 1970-79, n=1947.

**Fixed effects**	**Estimate ± SE**	**z-value**
**(Intercept)**	-2.13 ± 0.49	-4.38*
**Sex (Male)**	0.18 ± 0.17	1.10
**Month Birth**	0.05 ± 0.02	2.06*
**Birth Order (2rd-3rd born)**	-0.05 ± 0.20	-0.26
**Birth Order (4th+ born)**	-0.82 ± 0.29	-2.81*
**Mother's Age**	-0.04 ± 0.54	-0.08
**Mother's Age^2**	0.27 ± 0.52	0.52
**Mother's origin: Time since capture**	-0.09 ± 0.07	-1.39
**Birth Cohort (1980-89)**	-0.25 ± 0.21	-1.23
**Birth Cohort (1990-99)**	-0.20 ± 0.23	-0.89
**Birth Cohort (2000-13)**	-1.38 ± 0.34	-4.03*
**Random effects**	**Variance ± SD**	
**Location**	1.09 ± 1.04	

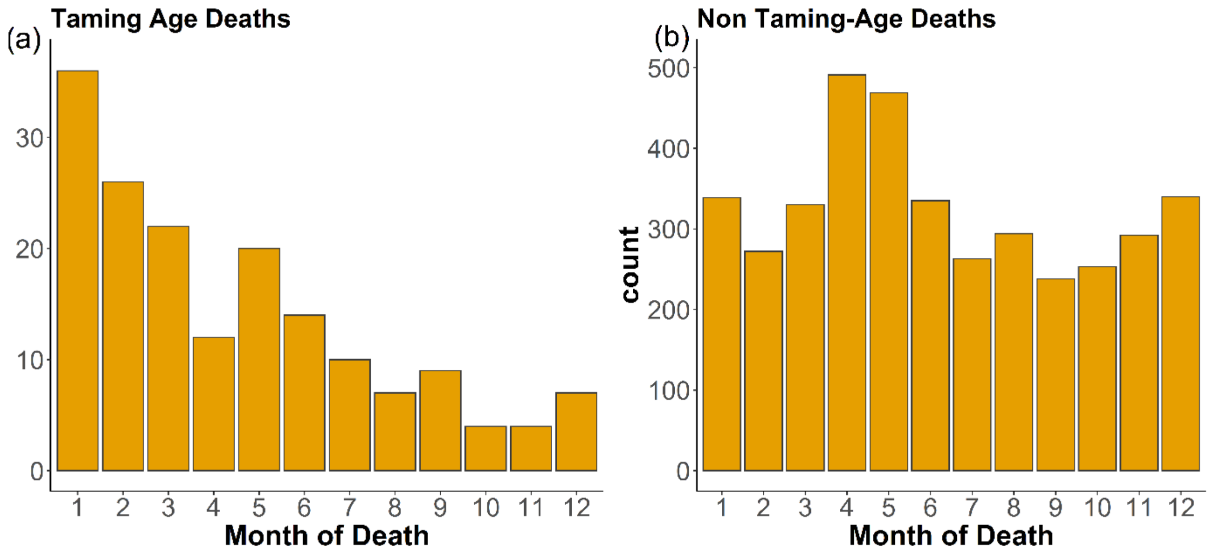

Figure S1. Distribution of deaths across in the year in (a) elephants of taming age (4.0-5.5 years, n=171) and (b) all other ages (n=3916). Month numbers correspond to months in a calendar year (ie. 1=January, 2=February etc).
