# Supplementary material for: Taming age mortality in semi-captive Asian elephants
Source: Sci Rep. 2020 Feb 5;10:1889. doi: 10.1038/s41598-020-58590-7 (PMC7002507; doi:10.1038/s41598-020-58590-7)
Supplement: Supplementary file 5 — Supplementary Information5. [file 41598_2020_58590_MOESM5_ESM.pdf]

Crawley, J. A. H., Lahdenperä, M., Min Oo, Z., Htut, W., Nandar, H., Lummaa, V. 2020. Taming age mortality in semi-captive Asian elephants.

Corresponding author:

Jennie A. H. Crawley

Department of Biology,

Natura Building, University of Turku,

Finland 20014

File list- 1 word file, 1 R script, 3 csv files, each labelled with a relevant file name:

1.Supplementary_Information.docx

2.Script_SR.R

3.Anon_Data_SR.csv

4.Anon_Captives_SR.csv

5.calf_mort_amboseli.csv

1.Supplementary_Information.docx contains information on the causes of death of 171 deceased calves in the paper, Table S1 model output of model including a quadratic mother's age term, and Figure S1 showing monthly distribution of deaths across the year of taming age elephants and elephants of other ages. All are referenced in the manuscript.

2.Script_SR.R contains the code to run the final model and produce the figures in the paper.

3.Anon_Data_SR.csv contains data on survival during taming ages (4.0-5.5 years) for 1,947 calves, with the columns needed to run the final model in Script.SR.R, and produce Figures 2a-c. Calf ID and mother ID have been changed for anonymity, and location has been given as categories.

4.Anon_Captives_SR.csv contains data on the survival of 2,962 captive born elephants, with their ID number changed for anonymity. This contains the columns needed to fit the survival curve from Script.SR.R, and produce Figures 1a-b.

5.calf_mort_amboseli.csv contains data on the probability of death for male and female calves over the first ten years in calves from the Amboseli wild African elephant population, taken from the life tables given in box 6.1 in The Amboseli Elephants book by Moss et al (2011), which is used to produce Figure 1c.
