# Supplementary figures and images for: Taming age mortality in semi-captive Asian elephants
Source: Sci Rep. 2020 Feb 5;10:1889. doi: 10.1038/s41598-020-58590-7 (PMC7002507; doi:10.1038/s41598-020-58590-7)

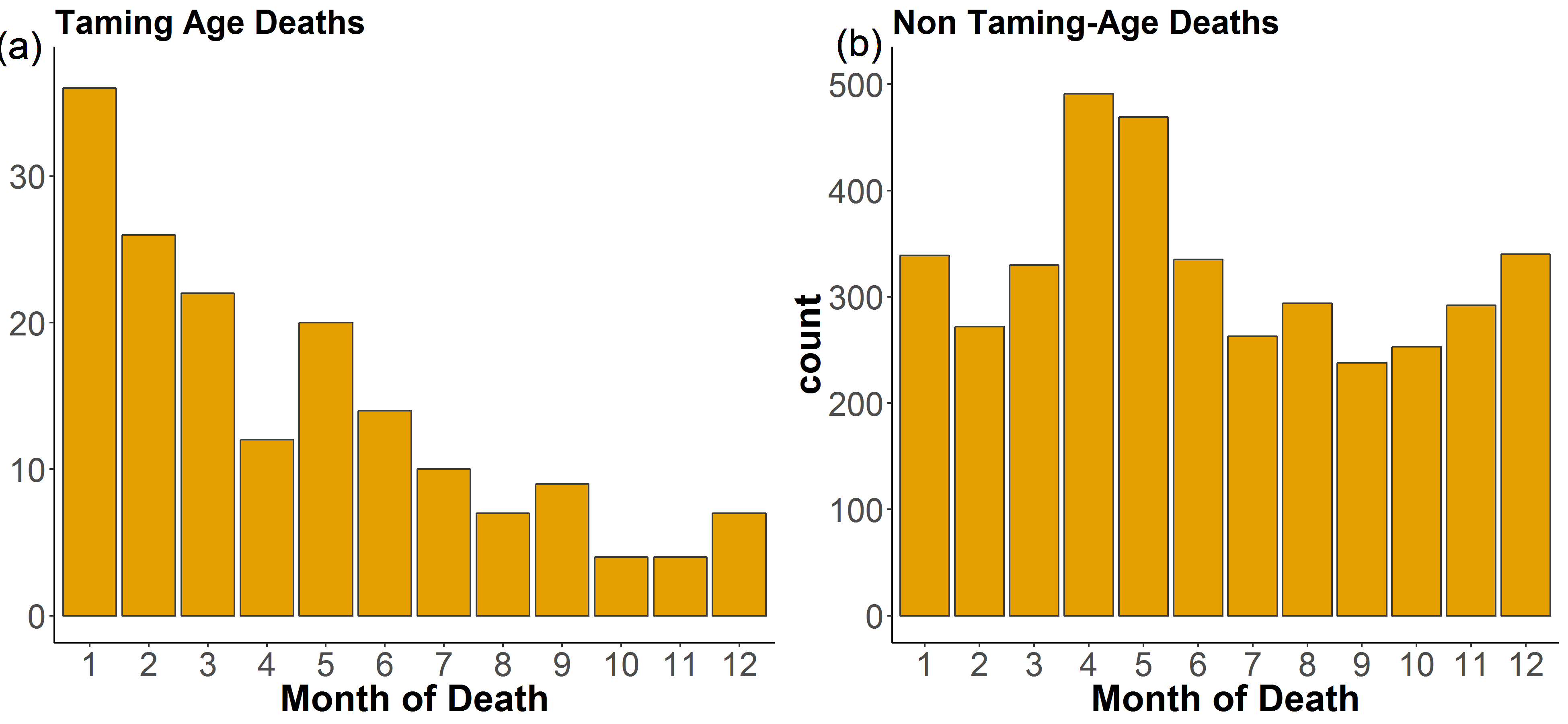

Supplement: Supplementary file 6 — Supplementary Information6. [file 41598_2020_58590_MOESM6_ESM.tiff]
